# A comparative psychological evaluation of a robotic avatar in Dubai and Japan

**DOI:** 10.3389/frobt.2024.1426717

**Published:** 2025-01-07

**Authors:** Hiroko Kamide, Yukiko Horikawa, Moe Sato, Atsushi Toyoda, Kurima Sakai, Takashi Minato, Takahiro Miyashita, Hiroshi Ishiguro

**Affiliations:** ^1^ The Center for Interdisciplinary Studies of Law and Policy, Graduate School of Law, Kyoto University, Kyoto, Japan; ^2^ Advanced Telecommunications Research Institute International (ATR), Kyoto, Japan; ^3^ The Graduate School of Engineering Science, Osaka University, Osaka, Japan; ^4^ Guardian Robot Project, National Research and Development Agency (RIKEN), Kyoto, Japan

**Keywords:** human-robot interaction, cultural comparison, avatar, Dubai, Japan

## Abstract

**Introduction:**

This study focused on the psychological evaluation of an avatar robot in two distinct regions, Dubai in the Middle East and Japan in the Far East. Dubai has experienced remarkable development in advanced technology, while Japan boasts a culture that embraces robotics. These regions are distinctively characterized by their respective relationships with robotics. In addition, the use of robots as avatars is anticipated to increase, and this research aimed to compare the psychological impressions of people from these regions when interacting with an avatar as opposed to a human.

**Methods:**

Considering that avatars can be presented on screens or as physical robots, two methodologies were employed: a video presentation survey (Study 1, Dubai: n = 120, Japan: n = 120) and an experiment involving live interactions with a physical robot avatar (Study 2, Dubai: n = 28, Japan: n = 30).

**Results and discussion:**

Results from the video presentations indicated that participants from Dubai experienced significantly lower levels of discomfort towards the avatar compared to their Japanese counterparts. In contrast, during live interactions, Japanese participants showed a notably positive evaluation towards a Japanese human operator. The findings suggest that screen-presented avatars may be more readily accepted in Dubai, while humans were generally preferred over avatars in terms of positive evaluations when physical robots were used as avatars. The study also discusses the implications of these findings for the appropriate tasks for avatars and the relationship between cultural backgrounds and avatar evaluations.

## 1 Introduction

In the burgeoning field of human-robot interaction (HRI), cultural dimensions play a pivotal role in shaping perceptions, acceptance, and integration of robotic technologies into society. Robots that exhibit cultural sensitivity are likely to enhance human acceptance (Rau et al., 2009; Salem et al., 2014). In addition, roboticists and developers stand to benefit from considering the cultural foundations that shape user perceptions and preferences (Lim et al., 2021). Historically, the bulk of comparative cultural studies in HRI has juxtaposed the attitudes and reactions between East Asian and Western populations. For instance, Castelo and Sarvary (2022) revealed that Americans exhibit a decrease in comfort with humanlike robots, a response not mirrored among Japanese participants, highlighting the influence of anthropomorphic features on comfort levels across cultures. This finding has been theoretically examined by Kaplan (2004), suggesting that the conceptual differences in perceiving the relationship between artifice and nature—where Western societies tend to dichotomize them, whereas Japanese culture finds a monistic connection between them—have a profound cultural impact on the acceptance of robots. Further exploration into the realm of HRI cultural comparisons introduces pivotal works, such as Li et al. (2024), which examines how the human-like appearance of service robots affects consumer trust across different cultures between China and United States. Nomura et al. (2015) explore the differences in social acceptance of humanoid robots between Japan and the United Kingdom, while Nomura et al. (2008) offer a cross-cultural analysis involving Japan, Korea, and the United States, focusing on public assumptions about humanoid and animal-type robots. Kamide and Arai (2017), and Komatsu et al. (2021) contribute to understanding the nuances of perceived comfortableness and blame attributions in HRI across the United States and Japan.

Although studies comparing East Asian perspectives with those from Australia and Mexico also exist—highlighted by Haring et al. (2014) and Bartneck et al. (2005) respectively—comprehensive comparative research involving the Middle East, particularly Dubai, is scant. Mavridis et al. (2012) present a seminal exploration into the attitudes toward humanoid robots in the Middle East, yet the study lacks a direct comparative cultural lens, underscoring an academic gap in juxtaposing Middle Eastern attitudes with those of other regions, notably against the backdrop of Dubai’s rapid economic and technological advancements. Understanding the social integration of robots and advanced technologies in Dubai, a city experiencing rapid technological development, from a cultural perspective is crucial. This importance stems from several reasons. First, the unique blend of traditional values and futuristic ambitions in Dubai’s society plays a significant role in shaping the public’s reception of technological innovations. Additionally, cultural attitudes towards technology can influence the pace at which these innovations are adopted and integrated into daily life. Furthermore, considering cultural perspectives can aid in the development of technologies that are not only technologically advanced but also culturally sensitive, ensuring a smoother integration into the fabric of society. This approach can help mitigate potential social resistance and foster a more inclusive environment for technological advancement.

The significance of conducting a comparative study between Dubai and Japan cannot be overstated. This significance extends beyond merely addressing the research gap caused by the lack of cultural comparisons involving Dubai; it also unveils new dimensions in the cross-cultural study of HRI. Specifically, Japan’s robot culture, often noted for its high affinity with robots (e.g., MacDorman et al., 2009; Šabanović, 2014; Sakura, 2021), serves as an important benchmark for comparison. For instance, recent developments in Japan involve using actual robots as avatars to create accessible workplace environments, including the development of avatar robots that enable physically disabled individuals to work in cafes (Ogushi et al., 2023; Takeuchi et al., 2020). “Avatar” can refer to a fully independent artificial character or something that acts virtually on behalf of a user (Pan and Hamilton, 2018), and in this study, we focus on the latter interpretation. As robots increasingly permeate society and establish relationships with humans, the utilization of robots as avatars, which are more feasible than fully autonomous robots, necessitates an understanding of how they are perceived from the perspective of the general public in each culture, to align research and development efforts accordingly.

From this perspective, understanding the fundamental psychological impressions of robots used as avatars in Dubai and Japan—two culturally distinct regions within Asia—becomes a critical scientific endeavor in the current era. This study represents the first cultural comparative analysis of HRI in Dubai and Japan, aiming to pioneer an understanding of the basic psychological interactions between humans and robots used as avatars in these regions. Avatars can vary, including those represented on screens (e.g., Kang and Watt, 2013; Miao et al., 2022; Schuetzler et al., 2018) or those embodied by physical robots (e.g., Lee et al., 2009; Ogushi et al., 2023; Takeuchi et al., 2020; Watanabe and Ho, 2023), thus considering the differences in presentation modes is vital for practical implementations. It is true that screen-based avatars may, in some cases, be substituted by direct videos of remote users. However, avatars can offer unique advantages in facilitating private interactions, as individuals may feel more comfortable engaging with avatars in sensitive contexts (e.g., AVITA, 2023). Therefore, it is essential to empirically investigate the effectiveness of avatars in such scenarios. Furthermore, when considering cases that involve the provision of physical services (e.g., Ogushi et al., 2023; Takeuchi et al., 2020), it is equally important to focus on robot-based avatars. Therefore, this research initially collects preliminary findings through video-based surveys, followed by field experiments using actual robots to conduct a comprehensive examination of HRI in these culturally diverse areas. By comparing the use of robots as avatars in workplaces in Dubai and Japan against traditional human roles, this study seeks to unveil unique insights into the acceptance and integration of robotic avatars and contribute new dimensions to the field of HRI.

Laying the groundwork for comparative cultural studies in HRI and exploring the intricate dynamics between Dubai and Japan, which represent distinct points on the cultural spectrum, is essential for understanding the unique perspectives each brings to technology integration. Dubai, in particular, has seen rapid technological development in recent years, with ambitions to become a completely paperless city through digitalization, underscored by significant government investment and support facilitating e-commerce development and technology adoption (Faccia et al., 2023). From a psychological perspective, considering culture, Dubai’s international milieu presents a stark contrast to Japan’s homogeneous society (Demelius, 2020; Miller et al., 2019). Psychological constructs like global orientation (Chen et al., 2016) indicate a tendency to embrace multicultural knowledge and languages while resisting assimilation into one’s own culture, suggesting that openness to different cultures could significantly impact the psychological perception of robots as avatars as new social entities. Given its diverse mix of nationalities and religions, Dubai might demonstrate a more favorable reception to newcomer robots as avatars in society compared to Japan, where cultural homogeneity underpins different expectations and levels of acceptance.

Conversely, the rapid technological advancement in Dubai is a relatively recent phenomenon compared to Japan, where robots have been familiar fixtures for many years. This suggests that robots as avatars in Dubai may still be too novel, not yet fully culturally assimilated. For instance, while smart home technologies, including those for the elderly like fall detection features, are gaining attention in Dubai as the elderly population increases, concerns about privacy and apprehension among Dubai’s elderly have been noted (Arar et al., 2021). In Japan, however, there’s a cultural continuity where distinctions between gods, humans, animals, and even inanimate objects like stones are not sharply delineated, suggesting that avatar robots could be more seamlessly integrated into the societal fabric (Kaplan, 2004). Indeed, many Japanese already perceive robots as part of their daily lives (Suwa et al., 2020), and robot technology for elderly care has advanced significantly (Tanioka, 2019). Considering the dynamics of Dubai’s technological growth and multiculturality versus Japan’s entrenched robot culture and monoculturality, various factors could influence the psychological evaluation of robots working as avatars. This study aims to meticulously explore these aspects through surveys using videos and field experiments with actual robots.

To obtain fundamental psychological evaluations, this study assesses basic cognitive dimensions such as warmth, competence, and discomfort towards robots functioning as avatars. These dimensions, foundational to human cognition, serve as a lens for evaluating robots in avatar roles. Drawing on the study by Carpinella et al. (2017), that introduced the Robotic Social Attributes Scale (RoSAS), this research explores how these attributes—‘warmth’ indicating approachability and friendliness, “competence” reflecting efficiency and skill, and ‘discomfort’ showing potential anxieties or concerns evoked by robots—are perceived differently between Dubai and Japan. Additionally, in examining whether robots used as avatars are perceived as social entities, this study considers cultural orientation, such as an openness to different cultures, and investigates the relationship of these cognitive dimensions across regions. This approach allows for a comprehensive understanding of how cultural backgrounds influence the psychological aspects attributed to robot avatars, paving the way for more culturally adapted HRI development.

## 2 Study 1: online survey using videos

In Study 1, we conducted an online survey to explore the levels of warmth, competence, and discomfort perceived by people living in Dubai and Japan towards a robot used as an avatar compared to an actual human. The study was particularly focused on the use of robots as avatars in receptionist roles, a job centered around human interaction. Reception tasks represent a typical occupation where humans working remotely can perform duties through avatars, replacing the need for a physical presence on-site. Therefore, we produced two types of videos: one featuring a female android robot, operated as an avatar by a person located remotely, performing reception duties, and another showing the human female operator herself. The two videos were presented in a randomized order to participants from Dubai and Japan, who then responded to a survey based on scales of warmth, competence, and discomfort towards each target. Furthermore, to gain insights into the cultural nuances of Dubai and Japan, the study incorporated questionnaire items of religious beliefs and nationality of participants from each region, in addition to individual differences in global orientation, within the same survey. The online survey was carried out by an online research company in Japan. This survey, as well as the subsequent experiment, were conducted in English in Dubai and in Japanese in Japan. This research was approved by the Ethics Review Committee of Advanced Telecommunications Research Institute International (ATR), with the approval number 23-530.

### 2.1 Methods

#### 2.1.1 Participants

Participants in this study consisted of individuals residing in either Dubai or Japan, totaling 240 participants. Specifically, 120 participants were from Dubai, with an equal gender distribution (50% men), and an average age of 37.90 years (SD = 8.95), ranging from 22 to 64 years. Another 120 participants were from Japan, also with an equal gender distribution (50% men), and an average age of 44.12 years (SD = 12.26), with ages ranging from 21 to 65 years. The demographics of participants further included years of residence in their respective locations. Participants in Dubai reported an average of 15.42 years of residence (SD = 11.00), with a range from 1 to 46 years. In contrast, participants in Japan reported a longer average residence time of 43.93 years (SD = 12.36), with the range spanning from 21 to 65 years. These figures suggest that while many residents of Dubai are immigrants from other countries, Japanese people tend to spend most of their lives in Japan.

Relatedly, all participants from Japan possessed Japanese nationality, contrasting sharply with the diverse nationalities observed among residents of Dubai. The breakdown of nationalities in Dubai was as follows: UAE (n = 65), Indian (n = 18), Filipino (n = 13), Pakistani (n = 7), British (n = 3), Egyptian (n = 3), Jordanian (n = 2), Lebanese (n = 2), and single instances from Algeria, an unspecified Asian country, Bangladesh, China, Ghana, Nepal, and Sudan. Regarding religious affiliations, the Japanese participants predominantly identified with Buddhism (n = 58), with smaller representations from Shinto (n = 7), Christianity (Protestant) (n = 1), Taoism (n = 1), and other unspecified beliefs (n = 29). Twenty-four Japanese participants preferred not to disclose their religious affiliation. In contrast, the religious landscape in Dubai was markedly more varied, with Islam (Sunni) (n = 54) and Islam (Shia) (n = 18) being prominent, followed by Christianity (Catholic) (n = 21), Christianity (Protestant) (n = 11), Hinduism (n = 12), and Buddhism (n = 1). A few individuals (n = 3) identified with other religious beliefs. As anticipated, the demographic profiles of our participants align with the cultural contexts of Dubai and Japan. Japan’s participants largely reflected a homogenous nationality with limited diversity in religious beliefs. Conversely, the participants residing in Dubai exhibited a broad spectrum of nationalities and practiced a diverse array of religions.

#### 2.1.2 Videos and measurements

**FIGURE 1:**
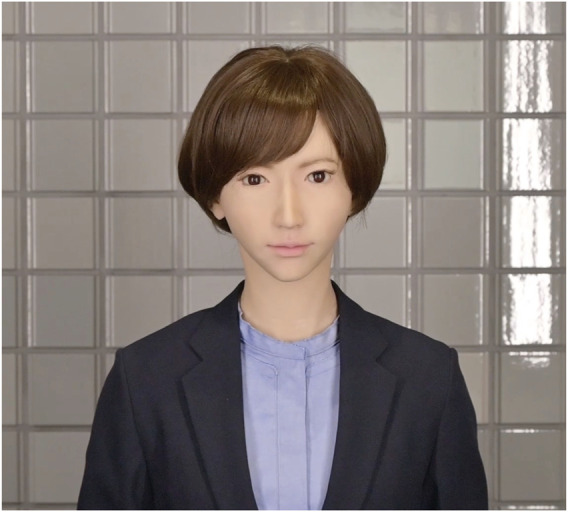
An android robot used as an avatar in the survey video.

In the creation of our video materials, both the robot as the avatar (Figure 1) and the human operator were depicted explaining the significance and utility of using robots as avatars. The robot used as an avatar in both Study one and Study two is the android ERICA (Glas et al., 2016; Minato et al., 2022). ERICA stands 166 cm tall in an upright position and features 44 active joints and 30 passive degrees of freedom. The robot’s movements are powered by pneumatic actuators. The video tailored for Dubai was produced in English, with a duration of 1 min and 50 s, while the version for Japan was made in Japanese, lasting 1 min and 41 s. The content provided by the avatar and the humans is parallel, the audio is played identically and identical scripts translated into the respective languages are used. In the video, both the avatar and the human operator began by introducing themselves, after which they proceeded to explain the applications of the avatar and the characteristics of the robot (see Supplementary Material).

After viewing the videos featuring either the avatar or the human, participants were asked to assess the target on scales of warmth, competence, and discomfort immediately following each viewing. Each scale consisted of six items. Responses were recorded on a nine-point Likert scale, ranging from 1 (definitely not associated) to 9 (definitely associated), to measure the degree of association participants attributed to each target in terms of warmth, competence, and discomfort. In the dimension of warmth, six items were employed to assess the perception: “Happy”, “Feeling”, “Social”, “Organic”, “Compassionate”, and “Emotional”. The Cronbach’s alpha for the avatar was calculated at 0.97, while for humans, it stood at 0.96. For the competence dimension, evaluation was based on six items: “Capable”, “Responsive”, “Interactive”, “Reliable”, “Competent”, and “Knowledgeable”. Here, the avatar’s Cronbach’s alpha was 0.96, compared to 0.95 for humans. The discomfort scale comprised six items: “Scary”, “Strange”, “Awkward, “Dangerous”, “Awful”, and “Aggressive”, with both avatars and humans registering a Cronbach’s alpha of 0.93.

Subsequently, participants responded to a scale designed to capture individual differences in global orientation (Chen et al., 2016). Global orientation represents individual differences in the psychological process of responding to globalization, defined as the emotional, behavioral, and cognitive reactions to cultural change. This scale is comprised of two sub-scales: “multicultural acquisition,” which indicates a proactive stance towards engaging with and acquiring new cultures, and “ethnic protection,” which represents a stance towards preserving one’s own culture. Originally a 25-item scale, it was condensed to 10 items for the Japanese translation (Iida et al., 2021), allocating five items to each sub-scale. Our study adopted this 10-item version for participants in both regions. The scale was a seven-point Likert scale ranging from 1 (strongly disagree) to 7 (strongly agree). Following exploratory factor analysis, one item that showed factor loadings of 0.40 or higher on both sub-scales was removed, leaving a total of five items for multicultural acquisition and four items for ethnic protection in the scale. The multicultural acquisition items included “I read books or magazines to obtain knowledge about other cultures,” “I learn customs and traditions of other cultures,” “I am happy to learn the history and geography of other cultures,” “I learn and speak languages other than my mother tongue,” and “I travel abroad to gain experiences with other cultures,” (Cronbach’s alpha was 0.93). The items of ethnic protection included “I find living in a multicultural environment very stressful,” “The ways that people of different cultural origins think and act often make me confused,” “I am worried that people from other cultures would not understand my ways of doing things,” and “Immigrants and ethnic minorities should forget their cultures of origin as much as possible for better adaptation to their new environment” (Cronbach’s alpha was 0.80).

### 2.2 Results and discussion

The descriptive statistics for each variable are presented in Table 1. In examining the sub-scales of global orientation, a one-way analysis of variance (ANOVA) revealed significant differences between Dubai and Japan in terms of multicultural acquisition (*F* (1,238) = 242.62, *p* < 0.001), with participants from Dubai scoring higher than their Japanese counterparts. This suggests that individuals residing in Dubai are more inclined to actively embrace languages, traditions, and customs from other cultures, whereas the propensity to positively engage with foreign cultures is less pronounced in Japan. On the other hand, no significant difference was observed in the ethnic protection, indicating that the extent to which individuals in both regions seek to preserve their own cultural identity does not differ significantly. Overall, as expected, Dubai appears to be more open to foreign cultures, reflecting its unique diversity characterized by the comings and goings of people from various religions and nationalities. On the other hand, Japan, being a more homogeneous society, seems to have a comparatively lower inclination towards actively adopting foreign cultures, suggesting a lower receptivity to other cultures.

**TABLE 1:** The descriptive statistics for each variable.

					Avatar			Human		
		Age	Multicultural acquisition	Ethnic protection	Warmth	Competence	Discomfort	Warmth	Competence	Discomfort
Dubai	M	37.90	5.84	3.73	5.28	5.81	4.10	6.25	6.48	4.00
	SD	8.95	1.17	1.75	2.56	2.43	2.48	2.23	2.14	2.54
Japan	M	44.12	3.33	3.89	3.64	4.81	4.67	4.54	5.21	3.80
	SD	12.26	1.32	0.99	1.68	1.82	1.61	1.61	1.59	1.50

Next, we examined the correlation between multicultural acquisition and ethnic protection, and their relationship with evaluations of warmth, competence, and discomfort towards the avatar and the human. The results of the correlation analysis are presented in Table 2, indicating that there was no significant correlation between multicultural acquisition and ethnic protection. This suggests that within our sample of Study 1, the inclination to positively embrace cultures other than one’s own and the tendency to cling to one’s native culture are not related. Importantly, multicultural acquisition was significantly positively correlated with warmth and competence attributed to both the avatar and the human, while it was negatively, albeit weakly, associated with discomfort towards the avatar. This implies that a lower openness to other cultures may result in higher discomfort evaluations towards avatars. The results indicated that perceptions of warmth towards the avatar were positively correlated with perceived competence of the avatar, suggesting that the warmer the avatar were perceived, the more competent the avatar regarded. Similarly, this pattern extended to the human target, where warmth was also positively associated with perceived competence. However, while there was a significant relationship between warmth and competence, these attributes did not correlate with evaluations of discomfort. Discomfort ratings for the avatar showed a positive correlation with discomfort ratings for the human, indicating a distinct dimension that might have a unique significance independent of the warmth and competence dimensions. This suggests that while warmth and competence are closely linked, discomfort may operate on a different affective or cognitive basis in social perceptions.

**TABLE 2:** Correlations between multicultural acquisition, ethnic protection, and the evaluation of warmth, competence, and discomfort towards the avatar and the human.

		Avatar			Human		
	Ethnic protection	Warmth	Competence	Discomfort	Warmth	Competence	Discomfort
Multicultural acquisition	n.s	0.42**	0.37**	−0.16*	0.46**	0.40**	n.s
Ethnic protection		0.20**	0.19**	0.34**	0.24**	0.17**	0.29**
Warmth (Avatar)			0.82**	n.s	0.68**	0.60**	n.s
Competence (Avatar)				n.s	0.58**	0.65**	n.s
Discomfort (Avatar)					n.s	n.s	0.66**
Warmth (Human)						0.85**	n.s
Competence (Human)							n.s

Note. *p < 0.05, **p < 0.01.

**FIGURE 2:**
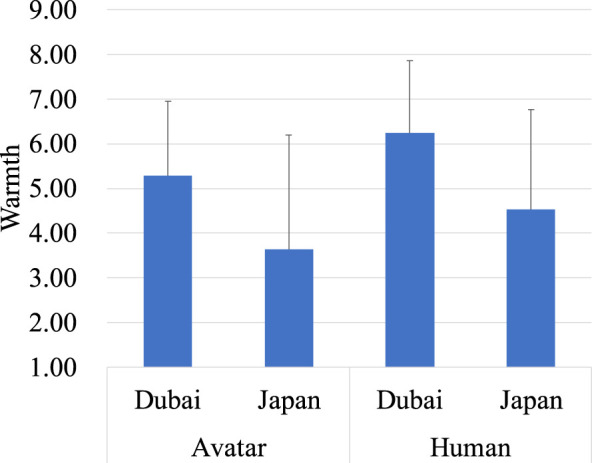
Evaluation of the avatar and the human on warmth.

Subsequently, a mixed ANOVA was conducted to investigate the effects of region (Dubai vs. Japan) and target type (avatar vs. human) on perceived warmth (Figure 2). The between-subjects factor was region, with two levels: Dubai and Japan. The within-subjects factor was target type, also with two levels: avatar and human. There was a significant main effect of region, *F* (1, 238) = 49.10, *p* < 0.001, indicating that perceived warmth was higher in Dubai (*M* = 5.77, *SD* = 1.70) compared to Japan (*M* = 4.09, *SD* = 1.70). Additionally, a significant main effect of target type was found, *F* (1, 238) = 65.69, *p* < 0.001, showing that the avatar (*M* = 4.46, *SD* = 2.31) were rated lower in warmth than the human (*M* = 5.39, *SD* = 2.12). The interaction effect between region and target type was not significant.

**FIGURE 3:**
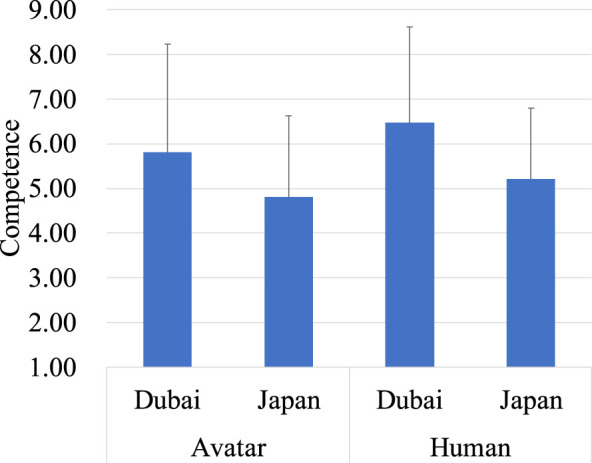
Evaluation of the avatar and the human on competence.

Similarly, the analysis on competence revealed a significant main effect of region, *F* (1, 238) = 23.22, *p* < 0.001, indicating that perceived competence was higher in Dubai (*M* = 6.14, *SD* = 0.17) compared to Japan (*M* = 5.01, *SD* = 0.17). Furthermore, the main effect of the target type was significant, *F* (1, 238) = 22.59, *p* < 0.001, indicating that perceived competence was higher in the human (*M* = 5.85, *SD* = 1.99) compared to the avatar (*M* = 5.31, *SD* = 2.20). There was not a significant interaction effect between region and target type (Figure 3).

**FIGURE 4:**
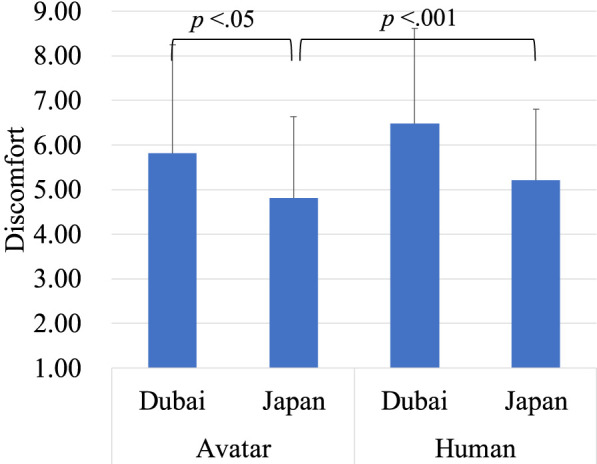
Evaluation of the avatar and the human on discomfort.

The analysis of discomfort revealed that the main effect of region was not significant, whereas the main effect of target was significant, *F* (1, 238) = 20.20, *p* < 0.01, indicating that the avatar (*M* = 4.39, *SD* = 2.11) were rated as more discomforting than the human (*M* = 3.90, *SD* = 2.08). There was a significant interaction between region and target type, *F* (1, 238) = 12.86, *p* < 0.001, indicating that the effect of target type on discomfort differed by region (Figure 4). Bonferroni *post hoc* tests showed that the avatar was rated as more discomforting in Japan (*M* = 4.67, *SD* = 1.61) than in Dubai (*M* = 4.10, SD = 2.48). Specifically, in Japan alone, the avatar (*M* = 4.67, *SD* = 1.61) was rated as more discomforting than the human (*M* = 3.80, *SD* = 1.50).

It can be posited that distinctive results were observed concerning discomfort. The tendency to feel discomfort or awkwardness towards the robot used as the avatar appears to be more pronounced in Japan than in Dubai. Given that discomfort signifies a sense of scariness or strangeness towards the presence of the target, this suggests that residents of Dubai may more readily accept the existence of robots used as avatars, without the discomfort impression for Japanese participants. Indeed, multicultural acquisition was significantly higher in Dubai than in Japan, correlating, albeit weakly, inversely with discomfort towards avatars. The tendency to be open to and seek to acquire cultures outside one’s own might also increase the likelihood of accepting new entities, such as robots serving as avatars, into society. This survey enabled the verification of fundamental insights into robots as avatars. However, given that this study was conducted using videos, further research involving direct interaction experiments with actual robots could deepen the understanding of the psychological evaluation of robots as avatars and their relationship with the cultures of Dubai and Japan. Although it is beyond the primary scope of this study, we also explored differences in the three impression evaluations—warmth, competence, and discomfort—of the robot as an avatar and the human operator with respect to nationality and religion. These results are included in the Supplementary Material (A similar analysis was not conducted for Study two due to the small sample size).

## 3 Study 2: interaction experiment with an actual robot as an avatar

In Study 1, utilizing online surveys with video content, it was found that individuals from Dubai exhibited a higher tendency to acquire multicultural knowledge, compared to those from Japan. It was also confirmed that, albeit weakly, lower levels of multicultural acquisition are associated with higher levels of discomfort towards the avatar. Furthermore, it was revealed that participants from Japan were particularly more likely to experience discomfort when watching the avatar than the human. Building on these findings, Study two employs an actual android robot to conduct similar receptionist tasks. The android robot as the avatar will be positioned at the entrance of the research facility in both Dubai and Japan, where the avatar will interact with the general public under the control of the operator woman. Following these interactions, participants will evaluate the robot as the avatar on warmth, competence, and discomfort, mirroring the methodology of Study 1. Additionally, the video of the woman who actually operated the robot will be shown in a video on a tablet, and the impressions will also be assessed on warmth, competence, and discomfort. Assessments of cultural orientation will also be conducted. In Study 2, by examining psychological assessments during interactions with the actual robot as the avatar, taking into account the results of Study 1, we aim to gain insights into the different modes of avatar usage.

### 3.1 Methods

The experimental procedure was largely consistent between Dubai and Japan, yet the recruitment methods for participants differed. In Dubai, experimenters approached passersby near the experimental site, which was equipped with a robot at the reception area, inviting them to interact with the robot as part of the study. Those who agreed participated in the experiment. In contrast, in Japan, participants were recruited in advance by a company specializing in participant recruitment. The experiments were completed in Dubai first, enabling the recruitment of Japanese participants of similar ages and prior experience interacting with robots as avatars, comparable to those from Dubai (Table 3). Participants interacted individually with the robot serving as tha avatar in both Dubai and Japan.

**TABLE 3:** Frequency of interactions with avatars in Dubai and Japan.

	Dubai		Japan	
	n	%	n	%
This is the first time	22	78.6	21	70
This is the second time	1	3.6	3	10
This is the third time	4	14.3	4	13.3
This is the fourth time	0	0	1	3.3
This is the fifth time or more	1	3.6	1	3.3
Total	28	100	30	100

#### 3.1.1 Participants

Participants consisted of 28 individuals in Dubai (average age = 32.88 years, *SD* = 8.79, age range = 21–51 years) including 13 men and 15 women, with missing age values for one man and two women. In Japan, the study included 30 participants (average age = 34.97 years, *SD* = 9.45, age range = 21–49 years), with an equal gender distribution of 15 men and 15 women. Additionally, the average length of residency was 12.00 years (*SD* = 10.70) in Dubai and 34.97 years (*SD* = 9.44) in Japan, with Japanese participants typically having resided in Japan their entire lives. Similar to Study 1, Dubai exhibited greater diversity in nationality and religion compared to Japan. In Japan, 29 participants held Japanese nationality, and one held South Korean nationality. In contrast, the nationalities represented in Dubai included UAE (n = 10), Filipino (n = 5), Russian (n = 3), American (n = 2), Indian (n = 2), Azerbaijani (n = 1), Italian (n = 1), Jordanian (n = 1), Lebanese (n = 1), Nepalese (n = 1), and Pakistani (n = 1). Regarding religious affiliations, the Japanese participants predominantly identified with Buddhism (n = 13), with others reporting no religious affiliation (n = 8), other unspecified beliefs (n = 3), Shinto (n = 4), and one participant preferred not to disclose their religious affiliation. Conversely, in Dubai, the religious landscape was relatively varied: Christianity (Catholicism) (n = 4), Christianity (Protestant) (n = 2), Hinduism (n = 2), Islam (Shia) (n = 2), Islam (Sunni) (n = 9), and other unspecified beliefs (n = 5).

#### 3.1.2 Experimental procedures and measurements

**FIGURE 5:**
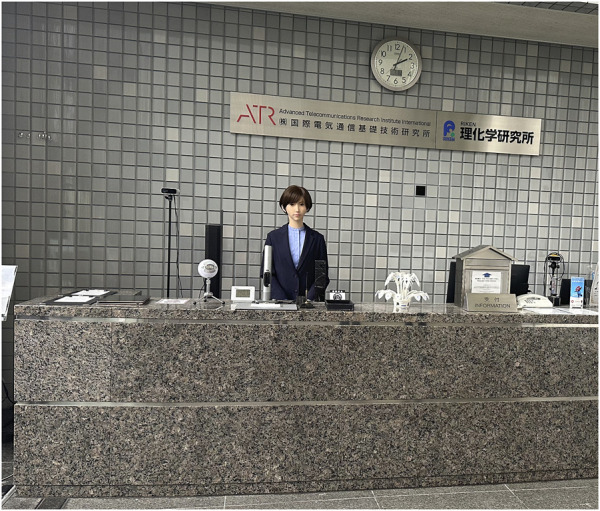
The android robot as the avatar Installed at the reception of ATR.

Specifically, the research institutions involved were the Dubai Future Labs in Dubai and the Advanced Telecommunications Research Institute International in Japan (Figure 5). As participants positioned themselves in front of the avatar, the operator initiated communication, first clarifying that the robot was not autonomous but was controlled by a human. Subsequently, she provided an overview of the research being conducted at either the lab in Dubai or the institute in Japan, followed by a question-and-answer session. During the question and answer sessions, common inquiries or comments received in both Dubai and Japan included, “Where are you remotely operating from?” and “I cannot make eye contact with the robot.” A notable comment from Dubai was, “The robot’s makeup is too light, making it appear less lively,” while in Japan, the question posed was, “Can you (the robot) move its hands?” The experimental session consisted of approximately 10 minutes of interaction, followed by about 3 minutes of impression evaluation conducted on a tablet.

Participants evaluated both the robot avatar they interacted with and the video of the operator based on dimensions of warmth, competence, and discomfort. The video displayed the actual operator speaking, and was presented without sound. As in Study 1, it would have been ideal to use a video of the operator that included audio information identical to the interaction with the avatar. However, since the content of the interaction naturally varies for each participant in the experiment, it was not feasible to prepare a pre-recorded video of the operator with audio that matched the live interaction. Furthermore, using a video with specific audio information raises concerns that the meaning conveyed by the audio could influence impression evaluations. Additionally, differences in participants’ interpretations of the audio might introduce uncontrolled biases, further affecting the reliability of the evaluations. Therefore, in this experiment, we opted to use a video of the operator without audio. Once they had a clear impression of her, they were asked to proceed with the impression evaluation. The sequence of evaluations between the avatar and the human was counterbalanced among participants. In Study 2, we employed a shortened version of the scales used in Study 1. This decision was made because, in experiments, participants are typically required to engage in more activities, such as receiving instructions and participating in live interactions, compared to surveys. As a result, the duration of the experiment is longer, increasing the potential for cognitive load. To minimize this load and avoid a decline in response quality due to reduced concentration, the number of questionnaire items was reduced. To ensure that each dimension was captured comprehensively rather than partially, we selected three items per dimension that were as distinct in meaning as possible. Furthermore, we calculated Cronbach’s alpha coefficients to verify that the reliability of the scales was not compromised by the reduction in items. For the dimension of warmth, three items were used (“Feeling”, “Happy”, “Organic”), yielding a Cronbach’s alpha of 0.92 for the avatar and 0.91 for the human. Competence was assessed with three items (“Knowledgeable”, “Capable”, “Competent”), with a Cronbach’s alpha of 0.92 for the avatar and 0.94 for the human. The discomfort scale consisted of three items (“Awful”, “Awkward”, “Dangerous”), with Cronbach’s alpha scores of 0.97 for the avatar and 0.81 for the human. The reliability coefficients demonstrated sufficiently high values. All items were rated using a nine-point Likert scale, consistent with the methodology in Study 1.

Additionally, the global orientation scale included a multicultural acquisition subsection with three items: “I learn customs and traditions of other cultures”, “I learn and speak languages other than my mother tongue”, and “I travel abroad to gain experiences with other cultures”, which had a Cronbach’s alpha of 0.82. The ethnic protection subsection was measured with three items: “I find living in a multicultural environment very stressful”, “The ways that people of different cultural origins think and act often make me confused”, and “I am worried that people from other cultures would not understand my ways of doing things”, resulting in a Cronbach’s alpha of 0.76. All items were rated using a seven-point Likert scale.

### 3.2 Results and discussion

Descriptive statistics for each variable are displayed in Table 4. Upon examining the sub-scales of global orientation, a one-way ANOVA was conducted. Significant differences were found between participants from Dubai and Japan in terms of multicultural acquisition (*F* (1,56) = 17.29, *p* < 0.001), with participants from Dubai scoring higher than their Japanese counterparts. This suggests that residents of Dubai are more proactive in learning about the customs, traditions, and languages of other cultures than residents of Japan, similar to findings from Study 1. Unlike in Study 1, significant difference was observed in the ethnic protection (*F* (1, 56) = 11.51, *p* < 0.001), where participants from Dubai scored lower than those from Japan. This indicates that Japanese residents are more likely to experience stress and confusion when dealing with other cultures compared to residents of Dubai. The findings reveal a clear contrast: people in Dubai are more inclined to embrace multicultural interactions and are less likely to feel perplexed or anxious about them, whereas in Japan, the tendency is the opposite.

**TABLE 4:** The descriptive statistics for each variable.

					Avatar			Human		
		Age	Multicultural acquisition	Ethnic protection	Warmth	Competence	Discomfort	Warmth	Competence	Discomfort
Dubai	M	32.88	5.75	3.30	5.98	6.82	3.46	6.54	6.80	3.20
	SD	8.79	0.99	1.39	2.26	1.93	1.93	1.90	1.94	1.73
Japan	M	34.97	3.84	4.70	5.78	6.78	2.41	7.92	8.04	1.44
	SD	9.45	2.23	1.72	2.05	1.90	1.24	0.86	1.13	0.75

In the subsequent analysis, the correlations between multicultural acquisition, ethnic protection, and the evaluations of warmth, competence, and discomfort towards the avatar and the human were examined (Table 5). In the sample of Study 2, multicultural acquisition and ethnic protection were significantly correlated, indicating that a stronger tendency to embrace multiculturalism was associated with a stronger inclination to protect one’s own ethnic culture. Furthermore, these two cultural orientations were not related to the impression ratings of the avatar and the human, suggesting that cultural attitudes may operate independently of social perceptions in this context. Additionally, although discomfort towards the human was not correlated with warmth and competence towards the avatar, other impression ratings were interrelated. The relationship among these variables appeared to differ depending on whether participants interacted with the avatar physically or observed videos, as in Study 1. In video observations, warmth and competence were correlated, and unrelated to discomfort. However, in live interactions, these variables seemed to be more interrelated, possibly indicating that high levels of warmth and competence generally correspond to lower levels of discomfort. However, since this study did not conduct a comparison between video observations in Study one and live interactions in Study two using the same sample who experienced both conditions, the discussions presented here remain speculative. Building on the findings of this research, it will be necessary to conduct a direct comparison in future studies.

**TABLE 5:** Correlations between multicultural acquisition, ethnic protection, and the evaluation of warmth, competence, and discomfort towards the avatar and the human.

		Avatar			Human		
	Ethnic protection	Warmth	Competence	Discomfort	Warmth	Competence	Discomfort
Multicultural acquisition	−0.40**	n.s	n.s	n.s	n.s	n.s	n.s
Ethnic protection		n.s	n.s	n.s	n.s	n.s	n.s
Warmth (Avatar)			0.77**	−0.45**	0.54**	0.64**	n.s
Competence (Avatar)				−0.42**	0.50**	0.72**	n.s
Discomfort (Avatar)					−0.31*	−0.45**	0.58**
Warmth (Human)						0.82**	−0.43**
Competence (Human)							−0.47**

Note. *p < 0.05, **p < 0.01.

**FIGURE 6:**
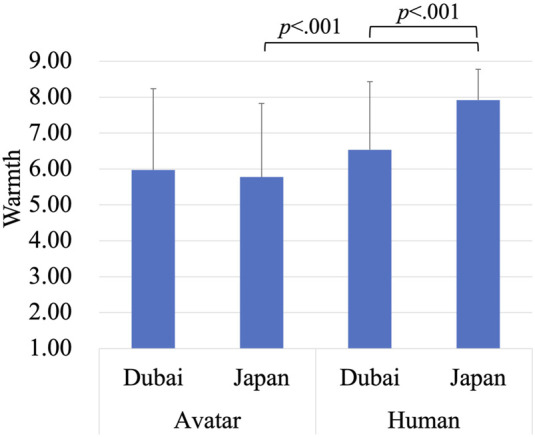
Evaluation of the avatar and the human on warmth.

A mixed ANOVA was conducted to investigate the effects of region (Dubai vs. Japan) and target type (avatar vs. human) on perceived warmth. The between-subjects factor was region, with two levels: Dubai and Japan. The within-subjects factor was target type, also with two levels: avatar and human. The main effect of region was not significant, indicating no difference in perceived warmth between Dubai and Japan. However, the main effect of target type was significant (*F(*1,56) = 36.87, *p* < 0.001), with the avatar (*M* = 5.87, *SD* = 2.14) rated as less warm compared to the human (*M* = 7.25, *SD* = 1.60). There was also a significant interaction between region and target type (*F* (1,56) = 12.67, *p* < 0.001). Specifically, in the human target condition, participants from Japan (*M* = 7.92, *SD* = 0.86) rated warmth higher than those from Dubai (*M* = 6.54, *SD* = 1.90). Moreover, in Japan, the human (*M* = 7.92, *SD* = 0.86) was rated as warmer than the avatar (*M* = 5.78, *SD* = 2.05). These findings of the interaction effect are illustrated in Figure 6.

**FIGURE 7:**
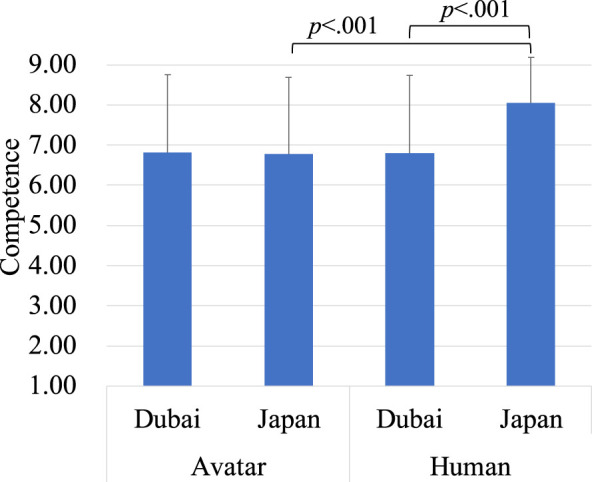
Evaluation of the avatar and the human on competence.

Similarly, in the analysis of competence, there was no significant main effect of region, but there was a significant main effect of target type (*F* (1,56) = 15.47, *p* < 0.001). This showed that perceived competence was higher for the human (*M* = 7.44, *SD* = 1.68) compared to the avatar (*M* = 6.80, *SD* = 1.90). There was also a significant interaction effect between region and target type. When the target was the human, competence ratings were higher in Japan (*M* = 8.04, *SD* = 1.13) than in Dubai (*M* = 6.80, *SD* = 1.94), and within Japan, the human (*M* = 8.04, *SD* = 1.13) was rated as more competent than the avatar (*M* = 6.78, *SD* = 1.90) (Figure 7). These results parallel those found in the warmth analysis, indicating that both warmth and competence ratings for Japanese operators were higher in Japan.

**FIGURE 8:**
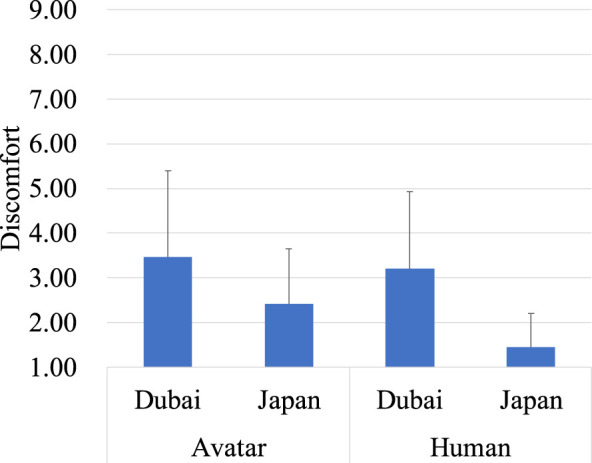
Evaluation of the avatar and the human on discomfort.

The analysis of discomfort revealed a significant main effect of region (*F* (1, 56) = 17.58, *p* < 0.01), with lower discomfort ratings in Japan (*M* = 1.93, *SD* = 0.79) compared to Dubai (*M* = 3.33, *SD* = 1.65). Additionally, there was a significant main effect of target type (*F* (1, 56) = 10.23, *p* < 0.01), with the avatar (*M* = 2.92, *SD* = 1.68) being rated as more discomforting than the human (*M* = 2.29, *SD* = 1.58). There was no significant interaction between region and target type (Figure 8). This indicates that discomfort towards both targets was generally lower in Japan than in Dubai, and in both regions, the avatar was found to be more discomforting than the human.

Overall, the actual human was perceived more positively than the robot serving as the avatar, being rated higher in warmth and competence and eliciting lower discomfort. This suggests that, at present, real humans are still more familiar and acceptable than realistic robots as avatars in both regions. Considering that androids like those used in this study have not yet become widespread as avatars, this finding appears rational. Moreover, the comparison between Dubai and Japan is distinct; people in Japan rated humans higher in warmth and competence compared to those in Dubai when the human was the target. After experiencing an interaction and then being introduced to the actual operator, Japanese participants might be more inclined to perceive their fellow Japanese as allies and to rate competence more highly. This suggests a potential cultural bias in favor of in-group members, which could influence the impression in social interactions among Japanese individuals.

## 4 General discussion

This study focused on Dubai, known for its rapid technological advancements, and Japan, recognized for its robot-friendly culture, aiming to elucidate foundational insights into how emerging avatar technologies are psychologically evaluated in both regions. Avatars are primarily classified into two modes: displayed on screens (e.g., Kang and Watt, 2013; Miao et al., 2022; Schuetzler et al., 2018) and utilizing actual robots (e.g., Lee et al., 2009; Ogushi et al., 2023; Watanabe and Ho, 2023). This study paid close attention to these modal differences, revealing distinctive psychological impressions related to each mode across the two regions. In the field of HRI, there has been a predominance of cultural comparisons between Western and East Asian cultures. However, there has been a notable gap in the comparison of cultures within the same Asian region, particularly between Dubai and Japan, each possessing distinctive technological cultures. This study addresses this gap by providing essential foundational knowledge on how avatars are psychologically perceived by residents in both regions. This represents an important effort to understand the cultural nuances that influence the adoption of avatar technologies in these uniquely characterized areas.

Considering both Study one and Study 2, whether on screen or in the physical robot, humans are perceived as warmer, more competent, and eliciting lower discomfort than avatars in both Dubai and Japan. Despite participants being aware that avatars are controlled by humans, the psychological discrepancy between avatars and their operators is an intriguing aspect. One conceivable reason for this could be the limited introduction of avatars in both Dubai and Japan, confined to specific contexts and settings. Additionally, the lack of everyday experience for most individuals in roles such as receptionists where avatars commonly operate might contribute. Reception duties typically involve highly significant and sophisticated management of customer experiences, as they represent the face of the organization or establishment (Bowditch and Garton, 2022), making it more likely for human agents to be perceived as appropriate for face-to-face customer interactions. Moreover, reception tasks often involve more flexible and interactive communication compared to rigid mechanical exchanges, potentially leading to the perception that real humans convey richer nonverbal cues than androids (Ishihara et al., 2021) and are easier to communicate with than avatars. Particularly, in the event of unforeseen accidents, avatars may be perceived as less useful due to their inability for immediate physical intervention, diminishing their utility in emergencies. Thus, the low level of familiarity with avatars and factors such as the nonverbal expressions and the level of tasks requested could potentially result in lower evaluations of warmth, competence, and higher discomfort for avatars compared to humans.

However, although humans were rated more positively than avatars, the results obtained from the two studies conducted using reception tasks are particularly suggestive from the perspective of task selection. Initially, the assumption that avatar receptionists would be feasible guided two studies, but it became evident that careful consideration of the characteristics of professions and tasks is essential. Reception tasks typically require a high degree of meticulousness, so when performed by avatars, simpler guidance tasks may be more readily accepted. Furthermore, the type of tasks performed by avatars may influence preferences for their appearance and mode, which could vary culturally. Additionally, by carefully considering these combinations, it is possible to explore the social value generated by avatar technology. As illustrated by cases in Japan (Ogushi et al., 2023; Takeuchi et al., 2020), the ground-breaking utilization of avatars for individuals unable to venture outdoors due to unavoidable circumstances represents a significant advancement. This not only signifies the utility of being able to work remotely but also generates inclusiveness of social participation, thereby possessing greater societal value. Thus, it is imperative to adopt a perspective that positions avatar technology within a broader dimension of societal value, connecting people and the utilization of avatars beyond mere usefulness to encompass wider societal value.

In examining the distinctive cultural differences between Dubai and Japan, notable insights emerge from two separate studies. According to the findings, residents in Dubai tend to perceive the avatar and the operator presented in videos with greater warmth and competence compared to their Japanese counterparts, while also rating discomfort significantly lower. This suggests that cultural predispositions influence how technologies, especially those representing human-like entities on screen, are received and emotionally processed. The evaluation of discomfort in the case of video presentations was particularly distinctive. The relative lack of discomfort with the avatar probably means that it is somewhat less difficult to accept its existence and co-exist with it. This is particularly intriguing given that the avatar especially on screen were less likely to evoke discomfort among Dubai respondents than among those from Japan. The implications of these findings are profound, suggesting that cultural openness in Dubai may extend to more readily embracing new technological forms as part of the cultural milieu, potentially viewing the limited existence of avatars ‘on screen’ as a new cultural entity.

However, a different pattern was observed in the second interaction study. Although people living in Dubai were still open to multiculturalism, this global orientation was not related to avatar or human impression ratings, rather the relationship between impression ratings was closer than in the case of the video observations. In both avatars and humans, warmth and competence were positively related, and these were negatively correlated with discomfort. That is, impressions may be more coherent in the case of direct interaction. Across both regions, the human was rated as warmer, more competent, and less discomforting than the avatar. Notably, Japanese participants perceived their fellow Japanese operator as particularly warm and competent, suggesting a characteristic positive evaluation towards compatriots. Focusing solely on the robots as the avatar, no regional differences in evaluations were observed. While there are discussions that Japan tends to feel a familiarity with robots (MacDorman et al., 2009; Šabanović, 2014; Sakura, 2021), this experiment did not demonstrate any significant differences with Dubai. It remains unclear whether this suggests cultural similarities in the psychological evaluations of avatar robots, or whether, in fact, cultural characteristics that are not yet visible might become apparent with longer-term use of avatars. Since the robots used in this study were developed in Japan, careful consideration should also be given to the appearance and functions of robots that might be more readily accepted within Dubai’s culture. Particularly for robots with human-like faces, it is necessary to consider the potential impact of cultural differences on facial expressions (Langlois et al., 2000; Perrett et al., 1994; Rhodes, 2006). Conducting comparative cultural studies of avatars from the perspective of non-verbal communication is also crucial.

The relationship between the human-like appearance of robots and cultural factors is particularly intriguing when examined through the lens of the uncanny valley phenomenon (Mori, 1970; Mori et al., 2012). It has been noted that as robots become increasingly human-like up to a certain point, they are perceived as more approachable. However, surpassing a specific threshold of human likeness often evokes a sense of eeriness, which only diminishes when the robot achieves a level of human likeness sufficient to be perceived as highly familiar. In discussions about interactions with robots, it has been suggested that this sense of eeriness is more closely related to the robot’s capabilities of physical experience than its intelligence or cognitive capabilities (Gray and Wegner, 2012). Furthermore, some studies propose that the uncanny valley may involve not one but two distinct valleys (Kim et al., 2022). Interestingly, increasing both the physical and psychological human-likeness of robots has been shown to decrease comfort levels among Americans, whereas it does not result in a similar decline among Japanese participants (Castelo and Sarvary, 2022). Expanding such investigations to include the Middle East through a cross-cultural perspective could provide valuable insights into the number and depth of uncanny valleys and the types of robots that contribute to their formation. Such findings would be instrumental in guiding the culturally adaptive implementation of robots into society, ensuring their acceptance across diverse cultural contexts.

This study represents a foundational attempt to compare the psychological impressions of the robot as the avatar between Dubai and Japan. Utilizing both video and robot interaction approaches, we have gleaned valuable insights, marking progress for cross-cultural HRI research, which has traditionally been conducted in Western and Far Eastern contexts. However, the study has several limitations. Firstly, the small sample size is a notable constraint. Dubai’s diverse international composition makes it difficult to fully capture the regional characteristics with the limited sample size used in this survey and experiment. Future replication studies are essential to validate our findings further. Additionally, the study involved only one type of robot. Avatar technology is rapidly evolving, and the diversity of characters displayed on screens is nearly limitless. The appearance and functionality of avatars likely have varying impacts on their impressions across different regions. As discussed, the type of tasks deemed suitable for avatars may also vary by culture, necessitating a more careful consideration of avatars and their applications across professions. Furthermore, while this study focused on global orientation by examining Dubai’s openness to other cultures in contrast with Japan, there are many other dimensions through which culture can be understood. Even within Asia, variations in individualism versus collectivism and religious differences must be explored in future research. Despite the limitations noted in this study, careful improvements are being made to further develop comparative cultural HRI research that considers the diversity of Asia. Another critical consideration is the specific competencies required of avatars. In this study, we did not provide participants with a clear explanation of the necessary skills or functionalities of the avatars, potentially leading to superficial evaluations. Future studies should carefully identify and communicate the required competencies before assessments. Additionally, it is crucial to investigate how the perceived mismatch between the robot’s lip movements and audio may have influenced impression evaluations. These aspects warrant further scrutiny. Importantly, while this study highlighted the video-based observation and live interaction modes of avatars, it did not conduct a direct comparison between the two. Such a direct comparison is necessary to better understand the unique characteristics of each mode. Future work will continue to explore the socialization of advanced technologies such as robots and avatars.

## Data Availability

The datasets presented in this article are not readily available because the ethical approval conditions do not allow them to be distributed among research teams. The analytical and framework matrices are available upon request from the corresponding author. Requests to access the datasets should be directed to kamide.hiroko.3y@kyoto-u.ac.jp.
