# Supplementary material for: A comparative psychological evaluation of a robotic avatar in Dubai and Japan
Source: Front Robot AI. 2025 Jan 7;11:1426717. doi: 10.3389/frobt.2024.1426717 (PMC11746044; doi:10.3389/frobt.2024.1426717)
Supplement: Supplementary file 1 [file DataSheet1.pdf]

Excluding nationalities and religions with small sample sizes, we analyzed the impact of nationality and religion on warmth, competence, and discomfort ratings for the robot as an avatar and the human operator. Using nationality or religion as independent variables and each impression dimension as dependent variables, we conducted an analysis of variance (ANOVA). The results for Study 1 are presented below. A similar analysis was not conducted for Study 2 due to the small sample size.

<Study 1: Analysis of Nationality>

1. **The relationship between nationality and the evaluation of the avatar on warmth**

- The main effect of nationality: $F(3,212)=24.62$, $p<.001$

Table 1. The evaluation of warmth for the avatar

Nationality	M	SD	N
Indian	6.14	2.64	18
Japan	3.64	1.68	120
Philippines	3.37	1.98	13
UAE	5.95	2.28	65

Table 2. Results of Tukey HSD multiple comparisons for warmth evaluations of the avatar

Comparison		Mean Difference	Standard Error	p-value	Lower Bound (95% CI)	Upper Bound (95% CI)
Indian	Japan	2.50	0.50	<.001	1.21	3.80
	Philippines	2.77	0.72	<.001	0.90	4.64
	UAE	0.19	0.53	0.98	-1.17	1.56
Japan	Indian	-2.50	0.50	<.001	-3.80	-1.21
	Philippines	0.26	0.58	0.97	-1.23	1.76
	UAE	-2.31	0.31	<.001	-3.10	-1.52
Philippines	Indian	-2.77	0.72	<.001	-4.64	-0.90
	Japan	-0.26	0.58	0.97	-1.76	1.23
	UAE	-2.57	0.60	<.001	-4.13	-1.01
UAE	Indian	-0.19	0.53	0.98	-1.56	1.17
	Japan	2.31	0.31	<.001	1.52	3.10
	Philippines	2.57	0.60	<.001	1.01	4.13

2. **The relationship between nationality and the evaluation of the avatar on competence**

- The main effect of nationality: $F(3,212)=29.32$, $p<.001$

Table 3. The evaluation of competence for the avatar

Nationality	M	SD	N

Indian	6.51	2.42	18
Japan	4.81	1.82	120
Philippines	4.44	2.63	13
UAE	6.18	2.14	65

Table 4. Results of Tukey HSD multiple comparisons for competence evaluations of the avatar

Comparison		Mean Difference	Standard Error	p-value	Lower Bound (95% CI)	Upper Bound (95% CI)
Indian	Japan	1.70	0.51	0.01	0.37	3.03
	Philippines	2.07	0.74	0.03	0.16	3.98
	UAE	0.33	0.54	0.93	-1.07	1.73
Japan	Indian	-1.70	0.51	0.01	-3.03	-0.37
	Philippines	0.37	0.59	0.92	-1.16	1.91
	UAE	-1.37	0.31	<.001	-2.18	-0.56
Philippines	Indian	-2.07	0.74	0.03	-3.98	-0.16
	Japan	-0.37	0.59	0.92	-1.91	1.16
	UAE	-1.74	0.62	0.03	-3.34	-0.15
UAE	Indian	-0.33	0.54	0.93	-1.73	1.07
	Japan	1.37	0.31	<.001	0.56	2.18
	Philippines	1.74	0.62	0.03	0.15	3.34

3. The relationship between nationality and the evaluation of the avatar on discomfort

- The main effect of nationality: $F(3,212)=3.77$, $p<.05$

Table 5. The evaluation of discomfort for the avatar

Nationality	M	SD	N
Indian	3.19	2.53	18
Japan	4.67	1.61	120
Philippines	3.46	2.13	13
UAE	4.43	2.52	65

Table 6. Results of Tukey HSD multiple comparisons for discomfort evaluations of the avatar

Comparison		Mean Difference	Standard Error	p-value	Lower Bound (95% CI)	Upper Bound (95% CI)
Indian	Japan	-1.49	0.51	0.02	-2.82	-0.15
	Philippines	-0.28	0.74	0.98	-2.20	1.64
	UAE	-1.24	0.54	0.10	-2.65	0.16
Japan	Indian	1.49	0.51	0.02	0.15	2.82

	Philippines	1.21	0.59	0.18	-0.33	2.75
	UAE	0.24	0.31	0.86	-0.57	1.06
Philippines	Indian	0.28	0.74	0.98	-1.64	2.20
	Japan	-1.21	0.59	0.18	-2.75	0.33
	UAE	-0.97	0.62	0.40	-2.57	0.64
UAE	Indian	1.24	0.54	0.10	-0.16	2.65
	Japan	-0.24	0.31	0.86	-1.06	0.57
	Philippines	0.97	0.62	0.40	-0.64	2.57

4. The relationship between nationality and the evaluation of the human on warmth

- The main effect of nationality: $F(3,212)=27.57$, $p<.001$

Table 7. The evaluation of warmth for the human

Nationality	M	SD	N
Indian	6.33	2.25	18
Japan	4.54	1.61	120
Philippines	4.45	2.58	13
UAE	6.92	1.81	65

Table 8. Results of Tukey HSD multiple comparisons for warmth evaluations of the human

Comparison		Mean Difference	Standard Error	p-value	Lower Bound (95% CI)	Upper Bound (95% CI)
Indian	Japan	1.80	0.45	<.001	0.62	2.97
	Philippines	1.88	0.65	0.02	0.19	3.58
	UAE	-0.58	0.48	0.62	-1.82	0.66
Japan	Indian	-1.80	0.45	<.001	-2.97	-0.62
	Philippines	0.09	0.52	1.00	-1.27	1.44
	UAE	-2.38	0.28	<.001	-3.09	-1.66
Philippines	Indian	-1.88	0.65	0.02	-3.58	-0.19
	Japan	-0.09	0.52	1.00	-1.44	1.27
	UAE	-2.47	0.55	<.001	-3.88	-1.06
UAE	Indian	0.58	0.48	0.62	-0.66	1.82
	Japan	2.38	0.28	<.001	1.66	3.09
	Philippines	2.47	0.55	<.001	1.06	3.88

5. The relationship between nationality and the evaluation of the human on competence

- The main effect of nationality: $F(3,212)=14.44$, $p<.001$

Table 9. The evaluation of competence for the human

Nationality	M	SD	N
Indian	6.55	2.12	18
Japan	5.21	1.59	120
Philippines	5.09	2.55	13
UAE	6.91	1.87	65

Table 10. Results of Tukey HSD multiple comparisons for competence evaluations of the human

Comparison		Mean Difference	Standard Error	p-value	Lower Bound (95% CI)	Upper Bound (95% CI)
Indian	Japan	1.34	0.45	0.02	0.16	2.51
	Philippines	1.46	0.65	0.12	-0.23	3.14
	UAE	-0.37	0.48	0.87	-1.60	0.87
Japan	Indian	-1.34	0.45	0.02	-2.51	-0.16
	Philippines	0.12	0.52	1.00	-1.23	1.47
	UAE	-1.70	0.28	<.001	-2.41	-0.99
Philippines	Indian	-1.46	0.65	0.12	-3.14	0.23
	Japan	-0.12	0.52	1.00	-1.47	1.23
	UAE	-1.82	0.54	0.01	-3.23	-0.42
UAE	Indian	0.37	0.48	0.87	-0.87	1.60
	Japan	1.70	0.28	<.001	0.99	2.41
	Philippines	1.82	0.54	0.01	0.42	3.23

6. The relationship between nationality and the evaluation of the human on discomfort

- The main effect of nationality: *n.s.*

Table 11. The evaluation of discomfort for the human

Nationality	M	SD	N
Indian	3.42	2.54	18
Japan	3.80	1.50	120
Philippines	3.44	2.08	13
UAE	4.31	2.75	65

<Study 1: Analysis of Religion>

1. The relationship between religion and the evaluation of the avatar on warmth

- The main effect of religion: $F(7,224)=6.94$, $p<.001$

Table 12. The evaluation of warmth for the avatar

Religion	M	SD	N
Buddhism	3.34	1.54	59
Christianity (Catholicism)	4.86	2.55	21
Christianity (Protestant)	4.68	2.74	12
Hinduism	6.08	2.46	12
Islam (Shia)	6.19	2.34	18
Islam (Sunna)	5.28	2.54	54
Other	3.65	1.74	32
Prefer not to answer	4.10	1.93	24

Table 13. Results of Tukey HSD multiple comparisons for warmth evaluations of the avatar

	Comparison	Mean Difference	Standard Error	p-value	Lower Bound (95% CI)	Upper Bound (95% CI)
Buddhism	Christianity (Catholicism)	-1.52	0.55	0.10	-3.19	0.15
	Christianity (Protestant)	-1.34	0.68	0.50	-3.42	0.74
	Hinduism	-2.74	0.68	0.00	-4.82	-0.67
	Islam (Shia)	-2.85	0.58	<.001	-4.61	-1.08
	Islam (Sunna)	-1.94	0.40	<.001	-3.18	-0.70
	Other	-0.31	0.47	1.00	-1.75	1.13
	Prefer not to answer	-0.76	0.52	0.83	-2.35	0.83
Christianity (Catholicism)	Buddhism	1.52	0.55	0.10	-0.15	3.19
	Christianity (Protestant)	0.18	0.78	1.00	-2.20	2.55
	Hinduism	-1.23	0.78	0.76	-3.60	1.15
	Islam (Shia)	-1.33	0.69	0.53	-3.44	0.78
	Islam (Sunna)	-0.42	0.55	1.00	-2.11	1.27
	Other	1.21	0.60	0.48	-0.63	3.06
	Prefer not to answer	0.76	0.64	0.94	-1.20	2.72
Christianity (Protestant)	Buddhism	1.34	0.68	0.50	-0.74	3.42
	Christianity (Catholicism)	-0.18	0.78	1.00	-2.55	2.20
	Hinduism	-1.40	0.88	0.75	-4.08	1.28

	Islam (Shia)	-1.50	0.80	0.57	-3.95	0.94
	Islam (Sunna)	-0.60	0.68	0.99	-2.69	1.50
	Other	1.03	0.73	0.85	-1.19	3.26
	Prefer not to answer	0.58	0.76	0.99	-1.74	2.90
Hinduism	Buddhism	2.74	0.68	0.00	0.67	4.82
	Christianity (Catholicism)	1.23	0.78	0.76	-1.15	3.60
	Christianity (Protestant)	1.40	0.88	0.75	-1.28	4.08
	Islam (Shia)	-0.10	0.80	1.00	-2.55	2.35
	Islam (Sunna)	0.81	0.68	0.94	-1.29	2.90
	Other	2.44	0.73	0.02	0.22	4.66
	Prefer not to answer	1.99	0.76	0.16	-0.34	4.31
Islam (Shia)	Buddhism	2.85	0.58	<.001	1.08	4.61
	Christianity (Catholicism)	1.33	0.69	0.53	-0.78	3.44
	Christianity (Protestant)	1.50	0.80	0.57	-0.94	3.95
	Hinduism	0.10	0.80	1.00	-2.35	2.55
	Islam (Sunna)	0.91	0.58	0.78	-0.88	2.69
	Other	2.54	0.63	0.00	0.60	4.47
	Prefer not to answer	2.09	0.67	0.04	0.04	4.14
Islam (Sunna)	Buddhism	1.94	0.40	<.001	0.70	3.18
	Christianity (Catholicism)	0.42	0.55	1.00	-1.27	2.11
	Christianity (Protestant)	0.60	0.68	0.99	-1.50	2.69
	Hinduism	-0.81	0.68	0.94	-2.90	1.29
	Islam (Shia)	-0.91	0.58	0.78	-2.69	0.88
	Other	1.63	0.48	0.02	0.17	3.10
	Prefer not to answer	1.18	0.53	0.33	-0.43	2.79
Other	Buddhism	0.31	0.47	1.00	-1.13	1.75
	Christianity (Catholicism)	-1.21	0.60	0.48	-3.06	0.63
	Christianity (Protestant)	-1.03	0.73	0.85	-3.26	1.19
	Hinduism	-2.44	0.73	0.02	-4.66	-0.22
	Islam (Shia)	-2.54	0.63	0.00	-4.47	-0.60
	Islam (Sunna)	-1.63	0.48	0.02	-3.10	-0.17
	Prefer not to answer	-0.45	0.58	0.99	-2.22	1.32
Prefer not to answer	Buddhism	0.76	0.52	0.83	-0.83	2.35
	Christianity (Catholicism)	-0.76	0.64	0.94	-2.72	1.20
	Christianity (Protestant)	-0.58	0.76	0.99	-2.90	1.74

	Hinduism	-1.99	0.76	0.16	-4.31	0.34
	Islam (Shia)	-2.09	0.67	0.04	-4.14	-0.04
	Islam (Sunna)	-1.18	0.53	0.33	-2.79	0.43
	Other	0.45	0.58	0.99	-1.32	2.22

2. The relationship between religion and the evaluation of the avatar on competence

- The main effect of religion: $F(7,224)=2.72$, $p<.05$

Table 14. The evaluation of competence for the avatar

Religion	M	SD	N
Buddhism	4.78	1.91	59
Christianity (Catholicism)	5.44	2.50	21
Christianity (Protestant)	5.69	2.57	12
Hinduism	6.39	2.57	12
Islam (Shia)	6.45	2.23	18
Islam (Sunna)	5.80	2.33	54
Other	4.78	1.70	32
Prefer not to answer	4.69	1.83	24

Table 15. Results of Tukey HSD multiple comparisons for competence evaluations of the avatar

	Comparison	Mean Difference	Standard Error	p-value	Lower Bound (95% CI)	Upper Bound (95% CI)
Buddhism	Christianity (Catholicism)	-0.65	0.54	0.93	-2.31	1.01
	Christianity (Protestant)	-0.91	0.68	0.88	-2.98	1.16
	Hinduism	-1.61	0.68	0.26	-3.67	0.46
	Islam (Shia)	-1.67	0.57	0.08	-3.43	0.09
	Islam (Sunna)	-1.01	0.40	0.19	-2.24	0.22
	Other	0.00	0.47	1.00	-1.43	1.43
	Prefer not to answer	0.09	0.52	1.00	-1.49	1.67
Christianity (Catholicism)	Buddhism	0.65	0.54	0.93	-1.01	2.31
	Christianity (Protestant)	-0.26	0.77	1.00	-2.62	2.10
	Hinduism	-0.95	0.77	0.92	-3.32	1.41
	Islam (Shia)	-1.02	0.69	0.82	-3.11	1.08
	Islam (Sunna)	-0.36	0.55	1.00	-2.04	1.32
	Other	0.66	0.60	0.96	-1.18	2.49
	Prefer not to answer	0.74	0.64	0.94	-1.21	2.69

Christianity (Protestant)	Buddhism	0.91	0.68	0.88	-1.16	2.98
	Christianity (Catholicism)	0.26	0.77	1.00	-2.10	2.62
	Hinduism	-0.69	0.87	0.99	-3.36	1.97
	Islam (Shia)	-0.76	0.80	0.98	-3.19	1.67
	Islam (Sunna)	-0.10	0.68	1.00	-2.19	1.98
	Other	0.91	0.72	0.91	-1.30	3.12
	Prefer not to answer	1.00	0.75	0.89	-1.31	3.31
Hinduism	Buddhism	1.61	0.68	0.26	-0.46	3.67
	Christianity (Catholicism)	0.95	0.77	0.92	-1.41	3.32
	Christianity (Protestant)	0.69	0.87	0.99	-1.97	3.36
	Islam (Shia)	-0.06	0.80	1.00	-2.50	2.37
	Islam (Sunna)	0.59	0.68	0.99	-1.49	2.68
	Other	1.61	0.72	0.34	-0.60	3.82
	Prefer not to answer	1.69	0.75	0.33	-0.61	4.00
Islam (Shia)	Buddhism	1.67	0.57	0.08	-0.09	3.43
	Christianity (Catholicism)	1.02	0.69	0.82	-1.08	3.11
	Christianity (Protestant)	0.76	0.80	0.98	-1.67	3.19
	Hinduism	0.06	0.80	1.00	-2.37	2.50
	Islam (Sunna)	0.66	0.58	0.95	-1.12	2.43
	Other	1.67	0.63	0.14	-0.25	3.60
	Prefer not to answer	1.76	0.67	0.15	-0.28	3.80
Islam (Sunna)	Buddhism	1.01	0.40	0.19	-0.22	2.24
	Christianity (Catholicism)	0.36	0.55	1.00	-1.32	2.04
	Christianity (Protestant)	0.10	0.68	1.00	-1.98	2.19
	Hinduism	-0.59	0.68	0.99	-2.68	1.49
	Islam (Shia)	-0.66	0.58	0.95	-2.43	1.12
	Other	1.02	0.48	0.40	-0.44	2.47
	Prefer not to answer	1.10	0.52	0.42	-0.50	2.70
Other	Buddhism	0.00	0.47	1.00	-1.43	1.43
	Christianity (Catholicism)	-0.66	0.60	0.96	-2.49	1.18
	Christianity (Protestant)	-0.91	0.72	0.91	-3.12	1.30
	Hinduism	-1.61	0.72	0.34	-3.82	0.60
	Islam (Shia)	-1.67	0.63	0.14	-3.60	0.25
	Islam (Sunna)	-1.02	0.48	0.40	-2.47	0.44
	Prefer not to answer	0.09	0.58	1.00	-1.68	1.85

Prefer not to answer	Buddhism	-0.09	0.52	1.00	-1.67	1.49
	Christianity (Catholicism)	-0.74	0.64	0.94	-2.69	1.21
	Christianity (Protestant)	-1.00	0.75	0.89	-3.31	1.31
	Hinduism	-1.69	0.75	0.33	-4.00	0.61
	Islam (Shia)	-1.76	0.67	0.15	-3.80	0.28
	Islam (Sunna)	-1.10	0.52	0.42	-2.70	0.50
	Other	-0.09	0.58	1.00	-1.85	1.68

3. The relationship between religion and the evaluation of the avatar on discomfort

- The main effect of religion: *n.s.*

Table 16. The evaluation of discomfort for the avatar

Religion	M	SD	N
Buddhism	4.43	1.63	59
Christianity (Catholicism)	3.61	2.13	21
Christianity (Protestant)	4.89	2.19	12
Hinduism	3.62	2.40	12
Islam (Shia)	4.03	2.81	18
Islam (Sunna)	4.31	2.60	54
Other	4.75	1.54	32
Prefer not to answer	5.19	1.48	24

4. The relationship between religion and the evaluation of the human on warmth

- The main effect of religion: $F(7,224)=11.09$, $p<.001$

Table 17. The evaluation of warmth for the human

Religion	M	SD	N
Buddhism	4.43	1.67	59
Christianity (Catholicism)	5.64	2.47	21
Christianity (Protestant)	4.79	2.52	12
Hinduism	7.44	1.63	12
Islam (Shia)	7.22	1.39	18
Islam (Sunna)	6.45	2.01	54
Other	4.48	1.80	32
Prefer not to answer	4.66	1.49	24

Table 18. Results of Tukey HSD multiple comparisons for warmth evaluations of the human

	Comparison	Mean Difference	Standard Error	p-value	Lower Bound (95% CI)	Upper Bound (95% CI)
Buddhism	Christianity (Catholicism)	-1.21	0.47	0.18	-2.66	0.24
	Christianity (Protestant)	-0.36	0.59	1.00	-2.17	1.45
	Hinduism	-3.01	0.59	<.001	-4.82	-1.21
	Islam (Shia)	-2.79	0.50	<.001	-4.33	-1.25
	Islam (Sunna)	-2.02	0.35	<.001	-3.09	-0.94
	Other	-0.05	0.41	1.00	-1.30	1.20
	Prefer not to answer	-0.23	0.45	1.00	-1.61	1.15
Christianity (Catholicism)	Buddhism	1.21	0.47	0.18	-0.24	2.66
	Christianity (Protestant)	0.85	0.67	0.91	-1.21	2.92
	Hinduism	-1.80	0.67	0.14	-3.87	0.26
	Islam (Shia)	-1.58	0.60	0.15	-3.41	0.25
	Islam (Sunna)	-0.80	0.48	0.70	-2.27	0.66
	Other	1.16	0.52	0.35	-0.44	2.76
	Prefer not to answer	0.98	0.56	0.65	-0.72	2.69
Christianity (Protestant)	Buddhism	0.36	0.59	1.00	-1.45	2.17
	Christianity (Catholicism)	-0.85	0.67	0.91	-2.92	1.21
	Hinduism	-2.65	0.76	0.01	-4.98	-0.32
	Islam (Shia)	-2.43	0.69	0.01	-4.56	-0.30
	Islam (Sunna)	-1.66	0.60	0.11	-3.48	0.17
	Other	0.31	0.63	1.00	-1.62	2.24
	Prefer not to answer	0.13	0.66	1.00	-1.89	2.15
Hinduism	Buddhism	3.01	0.59	<.001	1.21	4.82
	Christianity (Catholicism)	1.80	0.67	0.14	-0.26	3.87
	Christianity (Protestant)	2.65	0.76	0.01	0.32	4.98
	Islam (Shia)	0.22	0.69	1.00	-1.90	2.35
	Islam (Sunna)	1.00	0.60	0.70	-0.82	2.82
	Other	2.96	0.63	<.001	1.03	4.89
	Prefer not to answer	2.78	0.66	<.001	0.77	4.80
Islam (Shia)	Buddhism	2.79	0.50	<.001	1.25	4.33
	Christianity (Catholicism)	1.58	0.60	0.15	-0.25	3.41
	Christianity (Protestant)	2.43	0.69	0.01	0.30	4.56

	Hinduism	-0.22	0.69	1.00	-2.35	1.90
	Islam (Sunna)	0.77	0.51	0.79	-0.78	2.33
	Other	2.74	0.55	<.001	1.06	4.42
	Prefer not to answer	2.56	0.58	<.001	0.78	4.34
Islam (Sunna)	Buddhism	2.02	0.35	<.001	0.94	3.09
	Christianity (Catholicism)	0.80	0.48	0.70	-0.66	2.27
	Christianity (Protestant)	1.66	0.60	0.11	-0.17	3.48
	Hinduism	-1.00	0.60	0.70	-2.82	0.82
	Islam (Shia)	-0.77	0.51	0.79	-2.33	0.78
	Other	1.96	0.42	<.001	0.69	3.24
	Prefer not to answer	1.79	0.46	0.00	0.39	3.19
Other	Buddhism	0.05	0.41	1.00	-1.20	1.30
	Christianity (Catholicism)	-1.16	0.52	0.35	-2.76	0.44
	Christianity (Protestant)	-0.31	0.63	1.00	-2.24	1.62
	Hinduism	-2.96	0.63	<.001	-4.89	-1.03
	Islam (Shia)	-2.74	0.55	<.001	-4.42	-1.06
	Islam (Sunna)	-1.96	0.42	<.001	-3.24	-0.69
	Prefer not to answer	-0.18	0.50	1.00	-1.72	1.37
Prefer not to answer	Buddhism	0.23	0.45	1.00	-1.15	1.61
	Christianity (Catholicism)	-0.98	0.56	0.65	-2.69	0.72
	Christianity (Protestant)	-0.13	0.66	1.00	-2.15	1.89
	Hinduism	-2.78	0.66	<.001	-4.80	-0.77
	Islam (Shia)	-2.56	0.58	<.001	-4.34	-0.78
	Islam (Sunna)	-1.79	0.46	0.00	-3.19	-0.39
	Other	0.18	0.50	1.00	-1.37	1.72

5. The relationship between religion and the evaluation of the human on competence

- The main effect of religion: $F(7,224)=6.57$, $p<.001$

Table 19. The evaluation of competence for the human

Religion	M	SD	N
Buddhism	5.30	1.67	59
Christianity (Catholicism)	5.92	2.50	21
Christianity (Protestant)	5.71	2.09	12
Hinduism	7.14	1.84	12

Islam (Shia)	7.42	1.48	18
Islam (Sunna)	6.62	1.96	54
Other	4.98	1.78	32
Prefer not to answer	5.07	1.24	24

Table 18. Results of Tukey HSD multiple comparisons for competence evaluations of the human

	Comparison	Mean Difference	Standard Error	p-value	Lower Bound (95% CI)	Upper Bound (95% CI)
Buddhism	Christianity (Catholicism)	-0.62	0.46	0.88	-2.04	0.80
	Christianity (Protestant)	-0.41	0.58	1.00	-2.18	1.36
	Hinduism	-1.84	0.58	0.04	-3.61	-0.07
	Islam (Shia)	-2.12	0.49	<.001	-3.62	-0.62
	Islam (Sunna)	-1.33	0.34	0.00	-2.38	-0.27
	Other	0.31	0.40	0.99	-0.91	1.54
	Prefer not to answer	0.23	0.44	1.00	-1.13	1.58
Christianity (Catholicism)	Buddhism	0.62	0.46	0.88	-0.80	2.04
	Christianity (Protestant)	0.21	0.66	1.00	-1.81	2.23
	Hinduism	-1.22	0.66	0.59	-3.24	0.80
	Islam (Shia)	-1.50	0.59	0.18	-3.29	0.30
	Islam (Sunna)	-0.70	0.47	0.81	-2.14	0.73
	Other	0.94	0.51	0.60	-0.63	2.51
	Prefer not to answer	0.85	0.55	0.77	-0.82	2.52
Christianity (Protestant)	Buddhism	0.41	0.58	1.00	-1.36	2.18
	Christianity (Catholicism)	-0.21	0.66	1.00	-2.23	1.81
	Hinduism	-1.43	0.75	0.54	-3.71	0.85
	Islam (Shia)	-1.71	0.68	0.20	-3.79	0.37
	Islam (Sunna)	-0.92	0.58	0.77	-2.70	0.87
	Other	0.72	0.62	0.94	-1.17	2.62
	Prefer not to answer	0.64	0.65	0.98	-1.34	2.61
Hinduism	Buddhism	1.84	0.58	0.04	0.07	3.61
	Christianity (Catholicism)	1.22	0.66	0.59	-0.80	3.24
	Christianity (Protestant)	1.43	0.75	0.54	-0.85	3.71
	Islam (Shia)	-0.28	0.68	1.00	-2.36	1.80
	Islam (Sunna)	0.52	0.58	0.99	-1.27	2.30

	Other	2.15	0.62	0.01	0.26	4.05
	Prefer not to answer	2.07	0.65	0.03	0.09	4.04
Islam (Shia)	Buddhism	2.12	0.49	<.001	0.62	3.62
	Christianity (Catholicism)	1.50	0.59	0.18	-0.30	3.29
	Christianity (Protestant)	1.71	0.68	0.20	-0.37	3.79
	Hinduism	0.28	0.68	1.00	-1.80	2.36
	Islam (Sunna)	0.79	0.50	0.75	-0.73	2.31
	Other	2.43	0.54	<.001	0.79	4.08
	Prefer not to answer	2.35	0.57	0.00	0.61	4.09
Islam (Sunna)	Buddhism	1.33	0.34	0.00	0.27	2.38
	Christianity (Catholicism)	0.70	0.47	0.81	-0.73	2.14
	Christianity (Protestant)	0.92	0.58	0.77	-0.87	2.70
	Hinduism	-0.52	0.58	0.99	-2.30	1.27
	Islam (Shia)	-0.79	0.50	0.75	-2.31	0.73
	Other	1.64	0.41	0.00	0.39	2.89
	Prefer not to answer	1.55	0.45	0.01	0.18	2.92
Other	Buddhism	-0.31	0.40	0.99	-1.54	0.91
	Christianity (Catholicism)	-0.94	0.51	0.60	-2.51	0.63
	Christianity (Protestant)	-0.72	0.62	0.94	-2.62	1.17
	Hinduism	-2.15	0.62	0.01	-4.05	-0.26
	Islam (Shia)	-2.43	0.54	<.001	-4.08	-0.79
	Islam (Sunna)	-1.64	0.41	0.00	-2.89	-0.39
	Prefer not to answer	-0.09	0.49	1.00	-1.59	1.42
Prefer not to answer	Buddhism	-0.23	0.44	1.00	-1.58	1.13
	Christianity (Catholicism)	-0.85	0.55	0.77	-2.52	0.82
	Christianity (Protestant)	-0.64	0.65	0.98	-2.61	1.34
	Hinduism	-2.07	0.65	0.03	-4.04	-0.09
	Islam (Shia)	-2.35	0.57	0.00	-4.09	-0.61
	Islam (Sunna)	-1.55	0.45	0.01	-2.92	-0.18
	Other	0.09	0.49	1.00	-1.42	1.59

6. The relationship between religion and the evaluation of the human on discomfort

- The main effect of religion: *n.s.*

Table 19. The evaluation of discomfort for the human

Religion	M	SD	N
Buddhism	3.59	1.42	59
Christianity (Catholicism)	3.36	2.05	21
Christianity (Protestant)	4.21	2.20	12
Hinduism	3.67	3.13	12
Islam (Shia)	4.10	2.86	18
Islam (Sunna)	4.26	2.60	54
Other	3.60	1.72	32
Prefer not to answer	4.58	1.17	24
